# The History and Capabilities of Herbal Simples

**Published:** 1890-07-19

**Authors:** W. T. Fernie


### The History and Capabilities of Herbal Simples.

By W. T. Fernie, M.D.

(*Continued from page* 212.)

### XV.—CELANDINE—CELERY.

#### Celandine (the lesser).

No lady in the country on a fine day, when early Spring bespangles every bank with brilliant golden stars of the lesser celandine, has a fair excuse for being late to five o'clock tea. For, punctually at that hour, the glossy yellow flowers close their showy petals until nine a.m. on the following morning. This conspicuous herald of Spring comes into timely blossom on, or about St. Perpetua's day, March 7, and it belongs to the order of buttercups, ranunculaceæ. With its acrid juice, and that of other pungent plants belonging to the same tribe, beggars in England used to produce sores about their body for the purpose of exciting pity, and getting alms. They afterwards cured these sores by applying fresh mullein leaves to heal them. On the doctrine of signatures the lesser celandine has acquired the name of pilewort, by reason of small oval tubercles attached to its thin stringy roots, which have been supposed to represent and to cure piles. For a similar reason the plant also bears the name of Ranunculus Ficaria, from its curative use in the disease called ficus—"a red sore in the fundament"—Littleton, 1684. It has been practically proved that the whole plant, when bruised, and made into an ointment with fresh lard, is really useful for healing piles; as also when employed to the part in the form of a poultice or hot fomentation. "There be likewise those who thinke that if the herbe be but carried about one that hath the piles, the paine forthuith ceaseth." It has sometimes happened that the little white tubercles collected about the roots of the plant when washed bare by heavy rains, and lying free on the ground have given rise to a supposed shower of wheat. As it were to redeem this plant, with its handsome hopeful early Spring blossoms, from the vulgarity of base uses, the poet Wordsworth specially loved the lesser celandine, and tuned his lyre to sweetly sing its praises :—

"There is a flower that shall be mine,
'Tis the little Celandine;
I will sing, as doth behove—
Hymns in praise of what I love."

#### Celery.

Bread and cheese and kisses seem to go naturally together, but why celery accompanies cheese at the end of dinner it is not so easy to see. This is as much a puzzle as why sucking pig and prune sauce should be taken together; of which delicacies James Bloomfield Rush, the Norwich murderer, desired that plenty should be served for his supper the night before he was hanged, on April 20th, 1849. Our edible celery is a cultivated variety of the wild plant, or Smallage, this word being a corruption of small-ache, the *ch* being changed into *g*. Apium graveolens, the Smallage, or wild celery, grows abundantly in moist English ditches, or in water; its root being coarse, with a fetid smell, and unwholesome as food. It is an umbelliferous herb; and like many of this order, when transplanted and bleached, becomes aromatic and cordial, and excellent at table. But, more than this, celery may well take rank as a curative herbal simple. Dr. Pareira has shown us that it contains sulphur (a known preventive of rheumatism) as freely as do the cruciferous plants, mustard, and the cresses, &c. In 1879 Mr. Gibson Ward, then the president of the Vegetarian Society, wrote some letters to the *Times*, which commanded considerable attention, about celery as a food and a medicament. "Celery," said he, "when cooked, is a very fine dish, both as a nutriment and as a purifier of the blood." "I will not attempt to enumerate all the marvellous cures I have made with celery, lest medical men should be worrying me *en masse*. Let me fearlessly say that rheumatism is impossible on this diet, and yet English doctors in 1876 allowed rheumatism to kill three thousand six hundred and forty human beings, every death being as unnecessary as a dirty face." If all this be true, small wonder is it that the London populace buy celery so eagerly on market nights from hand barrows in the more busy thoroughfares! The Smallage plant is an Apium, or parsley; the great parsley being the Largeage, or Large-ache. Every parsley is also a selinon; and in this instance the middle *n* of the word has been changed to an *r*, making it—seliron; or, in Italian,—celeri. The root of the wild celery was reckoned by the ancients one of the five greater aperient roots, and was employed in their diet drinks. Lord Verulam says that witches used an ointment made from the juice of the Smallage at their incantations. The seeds of our sweet celery are carminative, and act on the kidneys. An admirable tincture may be made from the seeds, when bruised, with spirit of wine, of which tincture a teaspoonful may be taken three times a day in a little water. Smallage, and each other parsley, is said to be named Apium from apis, a bee, because bees affect these plants. But such cannot be the case for certain botanical reasons. The single diminutive flowers of the parsleys, as of other umbelliferous plants, have united themselves in umbels, or heads of blossom, so as to look large, and acquire importance; but the honey is contained still in each of the many pigmy open flat little corollæ: and roman-nosed bees scorn to visit these. Thus the parsley "at homes" are frequented only by plebeian gnats, thrips, ants, and small flies. And thus, likewise, it happens that these modest floral clusters display none but useful work-a-day neutral tints, warranted to wear well, and to wash until threadbare. for the *proletariat*; instead of assuming the brilliant blues, the warm reds, and the gay golden yellows of those richly-decorated corollæ which better serve to fascinate painted butterflies, and to allure Lepidopterous lordlings.

